# The Role and Reprocessing of Attitudes in Fostering Employee Work Happiness: An Intervention Study

**DOI:** 10.3389/fpsyg.2017.00028

**Published:** 2017-01-19

**Authors:** Paige Williams, Margaret L. Kern, Lea Waters

**Affiliations:** Centre for Positive Psychology, Melbourne Graduate School of Education, University of MelbourneParkville, VIC, Australia

**Keywords:** work happiness, attitudes, psychological capital, organization culture, organizational virtuousness

## Abstract

This intervention study examines the iterative reprocessing of explicit and implicit attitudes as the process underlying associations between positive employee attitudes (PsyCap), perception of positive organization culture (organizational virtuousness, OV), and work happiness. Using a quasi-experimental design, a group of school staff (*N* = 69) completed surveys at three time points. After the first assessment, the treatment group (*n* = 51) completed a positive psychology training intervention. Results suggest that employee PsyCap, OV, and work happiness are associated with one another through both implicit and explicit attitudes. Further, the Iterative-Reprocessing Model of attitudes (IRM) provides some insights into the processes underlying these associations. By examining the role and processes through which explicit and implicit attitudes relate to wellbeing at work, the study integrates theories on attitudes, positive organizational scholarship, positive organizational behavior and positive education. It is one of the first studies to apply the theory of the IRM to explain associations amongst PsyCap, OV and work happiness, and to test the IRM theory in a field-based setting. In applying attitude theory to wellbeing research, this study provides insights to mechanisms underlying workplace wellbeing that have not been previously examined and in doing so responds to calls for researchers to learn more about the mechanisms underlying wellbeing interventions. Further, it highlights the need to understand subconscious processes in future wellbeing research and to include implicit measures in positive psychology interventions measurement programs. Practically, this research calls attention to the importance of developing both the positive attitudes of employees and the organizational culture in developing employee work happiness.

## Introduction

A growing body of research that applies positive psychology (PP) to the organizational context has focused on individual and organizational factors that influence employee wellbeing at work. As a whole, studies suggest that employee subjective wellbeing has a positive impact on both employees and organizations. For employees, wellbeing has been related to their levels of engagement (Harter et al., 2003), organizational citizenship behaviors (LePine et al., 2002) relationships at work (Waters and Stokes, 2015), job satisfaction and commitment (Kern et al., 2015), and overall career success (Boehm and Lyubomirsky, 2008). For organizations, employee wellbeing is linked with greater customer satisfaction, productivity, presenteeism, and effort at work, and with less voluntary turnover, absenteeism, compensation claims, and sick days (e.g., Keyes, 2005; Wright and Bonett, 2007; Giardini and Frese, 2008; Pricewaterhouse Coopers, 2014). Further, there is a growing expectation from employees that organizations will take an active role in supporting their wellbeing, and this has become an important point of competitive advantage for many organizations in the employment market (Martin et al., 2005).

The question becomes what can organizations do to promote the wellbeing of their employees? The Inside-Out Outside-In model (IO-OI) (Williams et al., 2016a) proposes that factors “inside” the employee (e.g., internal attitudes) and factors “outside” the employee (e.g., organizational culture) influence work happiness, which is defined by Fisher (2010) as employees' perspectives on their engagement with work, commitment to the organization, and job satisfaction (For a full discussion on this model of work happiness, see Williams et al., 2016a).

Evidence suggests that reciprocal and synergistic associations exist between positive employee attitudes (PsyCap), perception of organizational culture (organizational virtuousness, OV) and work happiness (Williams et al., 2015). However, less is understood about *mechanisms* that underpin these relationships. Previous research has found some support for the processes of selective exposure and confirmation bias as underlying mechanisms for these associations (Williams et al., 2016a). The purpose of the current study is to explore the iterative reprocessing of attitudes as a possible underlying mechanism. In doing so, this study is the first to apply attitude theory and change models to understanding the mechanisms that underly workplace wellbeing and responds to calls for researchers to learn more about the mechanisms underlying PP interventions (Lyubomirsky and Layous, 2013).

This paper will first provide a brief overview of the Inside-out Outside-in model (Williams et al., 2016b); it will then look more closely at positive employee attitudes and propose a framework for examining positive attitudes at work. Next it will examine the stability and consciousness levels of attitudes and explore the Iterative-Reprocessing Model (IRM) of attitudes as an underlying mechanism through which work happiness is developed. Finally, the results of the study will be presented and discussed and implications for practice and future research will be highlighted.

### The Inside-out Outside-in model

**Figure 1:**
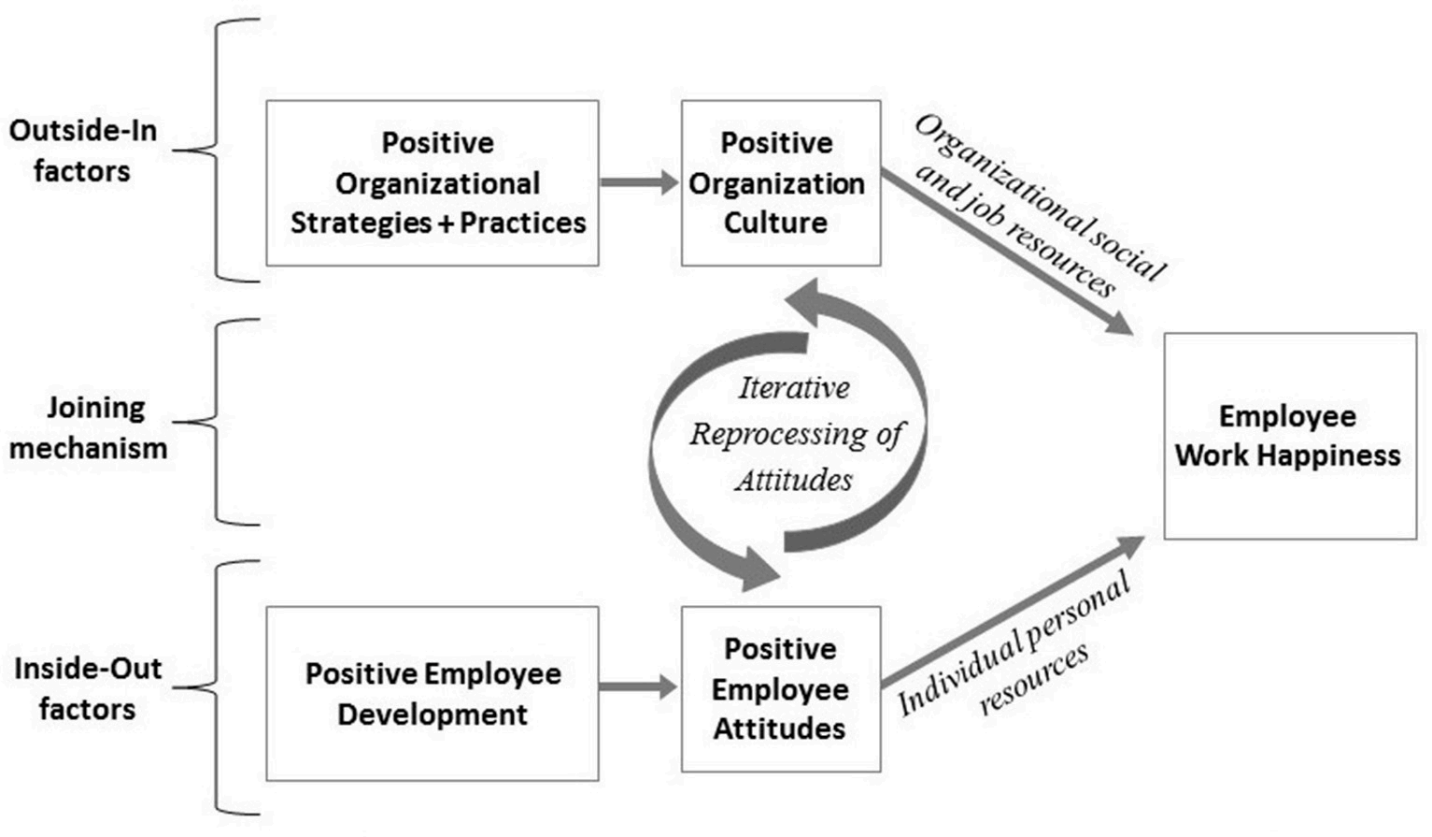
**The Inside-Out-Outside-In (IO-OI) model: A dual approach process model to developing work happiness (adapted from Williams et al., 2016b)**.

The Inside-out Outside-in (IO-OI) model (Williams et al., 2016b) is a dual approach process model that proposes that work happiness is influenced by factors “inside” the employee and factors “outside” of the employee (see Figure 1). Factors “inside” the employee are those that influence an employee's experience of work, but cannot be separated from the individual, for example attitudes, values, beliefs, emotions, and behaviors. Factors “outside” the employee are those that influence an employee's experience of work but that are discrete from the individual, e.g., organizational culture, job characteristics, manager/supervisor and the physical work environment. Interventions, then, might target factors “inside” individual employees, with effects ideally rippling across the organization (an inside-out approach), or might target factors “outside,” in the organization, with effects trickling down to impact the employees (an outside-in approach). For example, an “inside-out” approach might involve a positive employee development program that aims to develop positive employee attitudes to improve the employee's experience of work and increase their workplace wellbeing (see Figure 1). In contrast, an “outside-in” approach might use positive organizational strategies and practices, with the aim to develop a positive cultural environment in which employees work (see Figure 1). Thus, it is the *locus of the target of the change initiative* that determines whether the approach taken is “inside-out” or “outside-in” (Williams et al., 2016b).

While both inside-out and outside-in approaches enable and support work happiness, it is possible that a *combination* of the two provides the greatest benefits. Williams et al. (2015) conducted a longitudinal study exploring the association between employee PsyCap (i.e., positive attitudes—inside), perception of organizational virtuousness (OV) (i.e., positive culture—outside), and work happiness. Results suggested that within and across time, PsyCap and perception of OV were independently related to greater work happiness, but there was also some evidence of a synergistic effect between the inside and the outside factors. Similarly, in a sample of working adults, wellbeing was greatest when employees crafted their job (an inside-out approach) and were supported by their employers (an outside-in approach; Slemp et al., 2015). The IO-OI model also proposes underlying processes act as “joining mechanisms' that support the synergistic association between employee positive attitudes (inside factors), perceptions of organization culture (outside factors) and employee work happiness (see Figure 1). The current study examines the Iterative-Reprocessing Model (IRM) of attitudes as one such underlying process.

### Employee positive attitudes

Attitudes have been defined in various ways. Allport (1935) defines them as “mental and neural state of readiness” (p. 810), Eagly and Chaiken (1993) suggest that they are “a psychological tendency that is expressed by evaluating a particular entity with some degree of favor or disfavor” (p.1) and Bohner and Dickel (2011) propose they are “the evaluation of an object of thought” (p. 392) Attitudes can be positive (e.g., optimistic, hopeful) or negative (e.g., pessimistic, despairing). The focus in this study is the development and influence of positive attitudes, which are defined as “a positive evaluation of an object of thought” (Williams et al., 2016b). Positive attitudes of employees may support them in making positive evaluations about their job role, the behaviors of managers and colleagues and the organization culture, which may in turn influence their levels of work happiness (Williams et al., 2016b).

Studies of positive work attitudes have traditionally examined employee attitudes toward work (e.g., engagement, satisfaction, commitment). In such cases, the employee is required to make an evaluation about aspects of their work to create the attitude. For example, an employee may feel engaged with their work when they assess that they feel proud and happy in their role. We suggest that in addition to positive attitudes toward work, employees may also have positive attitudes *at* work, which are not specifically related toward their work, e.g., an employee with an optimistic attitude has positive expectations about the future (Williams et al., 2016a). We further suggest that employee positive attitudes *at* work may support more positive attitudes toward their work (Williams et al., 2016b). For example, an employee with a hopeful attitude may feel more satisfied with their job because they are making progress toward their work goals.

For the purposes of this study, positive attitudes are conceptualized as psychological capital (PsyCap, Luthans et al., 2006). In the positive organizational literature, PsyCap has been identified as a state of positive psychological development comprising four elements: hope, optimism, self-efficacy, and resilience (Luthans et al., 2006). Evidence suggests that these four elements synergistically lead to higher performance in the workplace than any of the elements individually (Luthans et al., 2010). PsyCap is proposed to be a “resource bank” that enables individuals to succeed in multiple life domains, including work, relationships and health (Hobfoll, 2002). Williams et al. (2016a) propose that PsyCap may also be considered a set of attitudes, due to a number of common processes and mechanisms between the two, including the cognitive, affective, and conative processes through which PsyCap influences wellbeing (Luthans et al., 2010; Youssef-Morgan and Luthans, 2014).

From this perspective, PsyCap provides an evidence-based framework that organizations can use to support organization members to develop four specific positive attitudes. A *hopeful* attitude is one where organization members have high levels of agency (goal-directed energy) and can generate pathways and plans to meet their goals (Snyder et al., 1996). An *optimistic* attitude comprises a positive expectation about the future (Scheier and Carver, 1985). A *self-efficacious* attitude involves “a conviction/confidence about one's abilities to mobilize the motivation, cognitive resources, or courses of action needed to successfully complete one's tasks and responsibilities” (Stajkovic and Luthans, 1998, p. 66). A *resilient* attitude is demonstrated through “positive adaptation in the context of significant adversity or risk” (Masten and Reed, 2002, p. 74).

Further supporting their conceptualization as attitudes, the four elements of PsyCap have been found to be open to change (Youssef and Luthans, 2007). Work-based PsyCap interventions have been shown to significantly increase attitudes of hope, optimism, efficacy, and resilience in organizational members (e.g., Luthans et al., 2008, 2010). For example, Luthans et al. (2006) developed a series of micro PsyCap interventions lasting from 1 to 3 hours and found that PsyCap significantly increased by 3%. As such, PsyCap is a valid model for organizations to use when developing positive attitudes in employees.

### Attitude stability

There are different views regarding the stability of attitudes over time. Some models, such as MODE (Motivation and Opportunity as Determinants, Fazio, 2007) and the meta-cognitive model (MCM; Petty et al., 2007), propose that attitudes are stored in long-term memory and are stable over time. Other models [e.g., Schwarz, 2007; Associative-Propositional Evaluation Model (APE), Gawronski and Bodenhausen, 2007], suggest that attitudes are formed in “real time,” and as such are impacted by the situational context and whatever information is accessible at that time. A third perspective combines these perspectives, suggesting that “current evaluations are constructed from relatively stable attitude representations” (Cunningham and Zelazo, 2007, p. 736). Together, these viewpoints suggest that research should take a more nuanced view of the stable vs. context-driven features of attitudes (Bohner and Dickel, 2011). The current study answers this call by examining the relationship between “real time” attitudes developed through training with more stable attitudes related to employee perceptions of organizational culture.

### Implicit vs. explicit attitudes

There is also evidence that attitudes operate at different levels of consciousness. *Explicit attitudes* are formed by individuals engaging in conscious reflection of factors that influence their evaluations and decisions. However, several studies have illustrated the limited accuracy of conscious reflection, as people have difficulty identifying what factors actually influence their attitudes and decisions (Nisbett and Wilson, 1977; Wilson, 2002). *Implicit attitudes* operate outside of an individual's full awareness and control. Although not easily reached through reflection, implicit attitudes have been shown to have powerful effects on behavior (Kay et al., 2004; Shantz and Latham, 2009). Implicit positive perceptions of others are associated with higher levels of organizational satisfaction, less cynicism, greater identification with their organization, and more positive peer-ratings of personality and popularity (Wood et al., 2010).

The cognitive processes underlying implicit and explicit attitudes may differ. Work by Fazio and Olson (2003) and Gawronski and Strack (2004) support the idea of a dual processing model of explicit and implicit cognition, in which the processes operate independently yet in parallel. These and other studies have shown that implicit processes are far less influenced by cognitive and motivational factors such as social desirability and evaluation than explicit cognitions (Roberts et al., 2006). Neurological research using functional magnetic resonance imaging provides further evidence for the dual processing model, by showing that implicit cognitions are processed in areas of the brain associated with automatic somatic and affective systems whereas explicit cognitions are processed in areas associated with deliberation and executive control (Lieberman, 2007).

Implicit processes are thought to be slow learning, in that they need a number of experiences to produce an aggregate knowledge base (McClelland et al., 1995), but fast acting, in that processing takes place in parallel and in millisecond cycles (Lord et al., 2010). As such, implicit processes require less cognitive resources to function. The knowledge-based, high-cognitive load work that dominates many twenty-first century organizations means that it is likely that implicit processing is extensive in organizational life (Johnson and Steinman, 2009). This has led to calls for research to examine “how people's explicit attitudes toward a company change through preceding shifts in implicit cognitions” (Uhlmann et al., 2012, p. 588). Further, implicit psychological constructs have been associated with a range of important organizational outcomes (Schultheiss, 2008) and specifically, implicit positive perceptions of others are associated with higher levels of organizational satisfaction in employees and greater identification with their organization (Wood et al., 2010). The current study examines the influence of implicit positive perceptions of organization culture and as such includes measures of both implicit and explicit perceptions of virtues in the organization culture (OV).

### Attitude development: the iterative-reprocessing model

**Figure 2:**
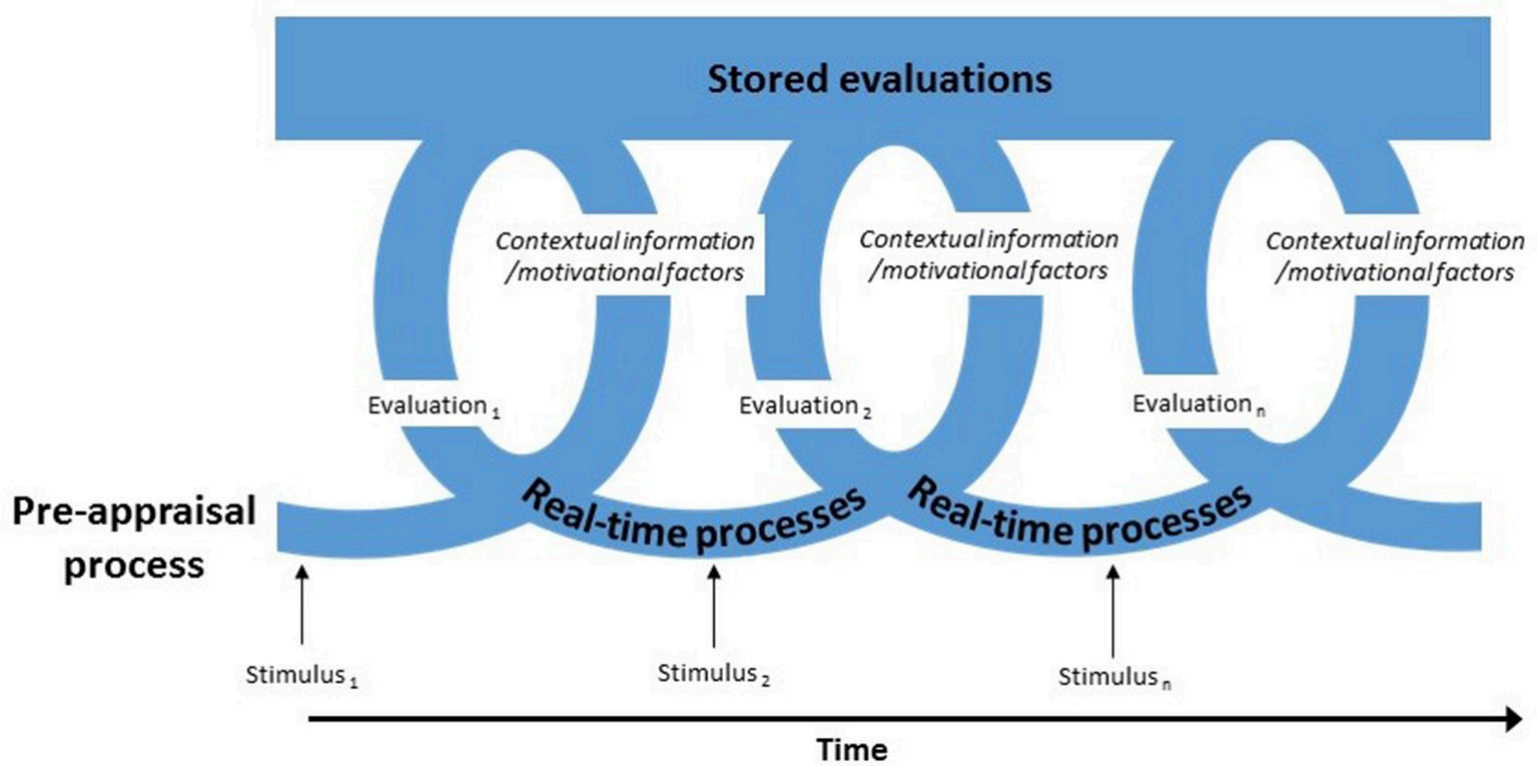
**The Iterative Cycle of Evaluation (adapted from Cunningham and Zelazo, 2007)**.

The Iterative-Reprocessing Model (IRM; Cunningham and Zelazo, 2007; Van Bavel et al., 2012) suggests that reflective processes play an important role in evaluation and attitude development. According to this model, objects of thought trigger an evaluative cycle through which lower-order, automatic (implicit) stored evaluations influence and are influenced by higher-order, reflective (explicit) real-time processes (see Figure 2). This cycle leads to a dynamic evaluation of attitude objects drawing on associations stored in memory and current information. As such, evaluation of an object of thought (attitude) involves the current processing state of the whole evaluative system, which is influenced by those elements of the stored attitude that are currently active and by the level of reflective real-time processing that takes place. Thus, in contrast to other models (Fazio and Olson, 2003; Gawronski and Strack, 2004) the IRM suggests that implicit and explicit cognitive and affective processes work in constant collaboration rather than as separate parallel systems. Further, the model proposes that the context, motivational state, and evaluative processes experienced immediately prior—known as the “pre-appraisal process”—also influences the evaluation process (see Figure 2). Each iterative cycle of evaluation is influenced by new contextual or motivational information, which creates a new evaluation of the attitude that considers finer attitude-object detail, the context and/or current goals (see Figure 2).

The number of times an attitude object goes through the iterative process depends on personal and situational variables such as cognitive ability, motivation, and opportunity (Fazio, 1990). Therefore, evaluations may be formed very quickly and remain stable for a time, or they may go through many iterative cycles over a long period of time and be continually altered and updated. Increasing reflective processes in individuals enables the more nuanced and/or goal congruent evaluations needed to navigate complex environments, self-regulate, and appraise abstract concepts such as virtues in the organization culture (Van Bavel et al., 2012). For example, consider the IRM process in a work context to explain how attitudes around a performance review can evolve. An employee has their annual performance review. Based on past experiences, their stored attitude associated with performance reviews is that they are negative, stressful experiences. However, in this specific situation, their manager adopts a positive, strengths-based approach to the performance review. The employee obtains feedback to improve their work, and the review has become less stressful. The actions of the manager create a change in context which means that a general attitude about reviews (that they are negative and stressful) and becomes more specific (reviews can be less stressful if approached in a positive way). Hence, while the automatic, stored (implicit) attitudes help to make quick assessments, these attitudes are dynamic and can be altered when the context or goals change through the influence of reflective (explicit) real-time processes. As such, the IRM suggests that there may be similar patterns for explicit and implicit evaluations, an assertion that is tested in the current study.

### The current study

Applying the IRM within the IO-OI framework, we propose that positive employee development through training can develop positive attitudes in employees (such as PsyCap; see Figure 1) which may trigger the iterative evaluation cycle (see Figure 2). Theoretically, training to develop PsyCap may provide organizational members with new goals and contextual information that enables them to develop increasingly positive attitudes—both explicit and implicit. These positive explicit and implicit attitudes may in turn support a *positive pre-appraisal* process (see Figure 2) which enables employees to see virtuous contextual and motivational cues in their organizational environment, thus increasing their perception of positive organizational culture and levels of work happiness (see Figure 1). This leads to hypotheses 1 and 2:

*H*1: Explicit attitudes of PsyCap and OV will be positively associated with work happiness. Specifically, (a) employee explicit PsyCap will be positively correlated with explicit OV, such that higher levels of PsyCap contribute to seeing more virtue in the organization culture, and greater virtue in the organization culture contributes to individual-level PsyCap. And (b) explicit PsyCap and explicit OV will be independently correlated with greater employee work happiness, both cross-sectionally and prospectively.
*H*2: Implicit attitudes of PsyCap and OV will be positively associated with work happiness. Specifically, (a) employee implicit PsyCap will be positively correlated with implicit OV, such that higher levels of implicit PsyCap contribute to seeing more virtue in the organization culture, and implicit OV contributes to implicit PsyCap. Further, (b) implicit PsyCap and implicit OV will be independently correlated with greater employee work happiness, both cross-sectionally and prospectively.

We further propose that the positive pre-appraisal process may lead to positive cycles of evaluation, as positive individual attitudes (PsyCap) are supported and reinforced by positive context and motivational cues in the organizational environment (OV), reflecting the iterative cycle of attitude development (Figure 2). This positive spiral may in turn lead to higher levels of employee happiness at work. Equally, it is surmised that the iterative process can work in a negative cycle, such that when the positive pre-appraisal process enabled through the development of specific positive attitudes (PsyCap) is *not* supported and reinforced by positive context and motivational cues in the organizational environment (OV), employee work happiness decreases. This leads to hypothesis 3:

*H*3: Reflecting the positive spiral of the IRM process as shown in Figure 2, work happiness will be higher when positive PsyCap attitudes are supported by a positive evaluation of the organization environment (OV) than when they are not.

Finally, the IRM argues that implicit and explicit processes work in collaboration rather than as separate systems; as such explicit and implicit attitudes would reflect similar changes over time. We test this through hypothesis 4:

*H4*: Based on the IRM, implicit and explicit evaluations will have similar patterns of change over time.

In this quasi-experimental study, 69 school staff members completed questionnaires at three time points (pre-intervention, immediately post-intervention, 8 weeks post-intervention). After the first assessment, 51 of the staff members completed a training program that focused on developing PsyCap, with the remaining staff members constituting a control group. Williams et al. (2016a) found that the intervention successfully developed PsyCap (positive employee attitudes in Figure 1). The current study focuses on the 51 employees who completed the training (i.e., the intervention group) to examine underlying processes to foster work happiness and specifically, whether through the development of implicit and explicit positive attitudes (PsyCap), the iterative reprocessing of attitudes was triggered.

## Methods

### Participants

A group of 51 employees (27 female, 24 male) of a large independent school in Victoria, Australia participated in this study; 27 were members of teaching staff (15 female, 12 male) and 24 were employed in non-teaching roles (12 female, 12 male). All 51 participants in the intervention group completed the questionnaire at time 1 and 2; 38 (73%) completed it at time 3. Participants were primarily between 35 and 64 years of age (25–34: *n* = 3, 35–44: *n* = 12, 45–54: *n* = 15, 55–64: *n* = 17, 65+: *n* = 4). The majority of the sample had been working at the school for less than 5 years (new this year: *n* = 25, 1–5 years: *n* = 19, 6–10 years: *n* = 5, 10+ years: *n* = 2). There were no significant differences between those who completed all three time points vs. those missing at time 3 in terms of demographics (age, gender, role in school, hours worked, tenure status) or in terms of time 1 implicit and explicit PsyCap, OV, or work happiness.

### Measures

Participants completed a survey, which combined pre-established scales measuring implicit and explicit PsyCap, employee perception of the virtues present in the organization (OV), and employee work happiness. At time points 1 and 2 the survey was completed by pen and paper and the results were input by the researcher. At time-point 3 the survey was completed on-line.

#### Positive attitudes

##### PsyCap

Explicit attitudes of PsyCap were measured through the 24-item self-rated PsyCap Questionnaire (PCQ; Luthans et al., 2007), which has been tested in a variety of samples, including service, manufacturing, high-tech, military, and education sectors and across national cultural settings. In the PCQ, the four factors (hope, optimism, resilience, and self-efficacy) are measured by six items each, which were adapted from other scales. Example items include: “There are lots of ways around any problem” (hope); “When things are uncertain for me at work, I usually expect the best” (optimism); “I usually take stressful things at work in stride” (resilience); and “I feel confident presenting information to a group of colleagues” (self-efficacy). Items were scored on a 6-point Likert scale from “strongly disagree” (1) to “strongly agree” (6), and were averaged together to represent the individual's level of PsyCap (24 items, Cronbach's α_t1_ = 0.89, α_t2_ = 0.85, α_t3_ = 0.88).

Implicit attitudes of PsyCap were measured using the I-PCQ (Harms and Luthans, 2012), which uses a semi-projective technique with written prompts followed by short questions. To elicit implicit attitudes related to PsyCap, respondents were presented with three situational prompts and asked to imagine stories relating to the prompt about a fictional character (not themselves). They were then asked to respond to construct-targeted questions about the stories they generated. The three prompts were based in an organizational context with one presenting a positive experience (i.e., “Someone has a new job”), one a negative experience (i.e., “Someone makes a mistake at work”), and the third an ambiguous experience (i.e., “Someone talks to their supervisor”). Harms and Luthans (2012) propose that the ambiguous prompt is similar to the Rorschasch Ink-Blot Test (Rorschach, 1921) in that it is particularly open to interpretation and projection by the respondent.

For each of the stories, participants indicated on a 7-point Likert scale how much each PsyCap factor represents the character (−3 = the opposite is very true of this character, 0 = irrelevant thought/feeling for this character, and +3 = very true of this character). The four factors of PsyCap were assessed as follows: “believing that they can accomplish their goal” (hope); “expecting good things to happen in the future” (optimism); “believing that they can bounce back from any setbacks that have occurred” (resilience); and “feeling confident and self-assured in their ability” (self-efficacy). Each prompt also included four filler items to help disguise the intent of the measure. The 12 items (the four PsyCap questions across the three stories) were averaged together to represent an overall Implicit PsyCap score (α_t1_ = 0.88, α_t2_ = 0.93, α_t3_ = 0.91).

#### Positive culture

##### Organizational virtuousness (OV)

The Organizational Virtuousness Scale (Cameron et al., 2004) was used to measure explicit OV. Work by Cameron et al. (2004) resulted in a five factor model, which suggests that organizational virtuousness (OV) comprises: (1) organizational forgiveness, (2) organizational trust, (3) organizational integrity, (4) organizational optimism, and (5) organizational compassion. Items include: “we try to learn from our mistakes here, consequently missteps are quickly forgiven” (forgiveness); “people trust the leadership of this organization” (trust); “this organization demonstrates the highest levels of integrity” (integrity); “we are optimistic that we will succeed, even when faced with major challenges” (optimism); and “this organization is characterized by many acts of caring and concern for other people” (compassion). Each of the 15 items was scored on a 6-point Likert scale from “strongly disagree” (1) to “strongly agree” (6), with higher scores indicating a greater perceived presence of that dimension of OV. The items were averaged together to represent an individual's explicit OV score (15 items, α_t1_ = 0.96, α_t2_ = 0.93, α_t3_ = 0.95).

While there is an existing measure of implicit PsyCap (I-PCQ) attitudes, there is not an existing measure of implicit attitudes of OV. We sought advice from Peter Harms (co-author of the I-PCQ) to construct an implicit OV measure. Using the three prompts from the I-PCQ, the existing measure was extended to include 10 items from the explicit organizational virtuousness scale (Cameron et al., 2004). For each of the stories, the five factors of OV were assessed by two items, such as “we forgive mistakes when they are acknowledged and corrected” (forgiveness) and “honesty and trustworthiness are hallmarks of this workplace” (trust). The 10 items across the three prompts were averaged together to represent an individual's implicit OV (30 items, α_t1_ = 0.96, α_t2_ = 0.98, α_t3_ = 0.97). Information regarding the development and initial psychometric testing for the implicit OV measure is provided in the Supplementary Materials.

#### Work happiness

Work happiness was defined using Fisher's (2010) model comprising (1) engagement with the work itself; (2) satisfaction with the job, including contextual features such as pay, co-workers, supervision, and environment; and (3) feelings of affective commitment to the organization as a whole. As a single existing measure of this model does not currently exist, three scales were used to represent these three factors. Engagement with work was measured using the 9-item Utrecht Work Engagement Scale (UWES-9; Schaufeli and Bakkar, 2003). Job satisfaction was measured with the 8-item Job in General Scale (JIG; Russell et al., 2004). Affective commitment to the organization was measured using a 9-item positively phrased version of the 15-item Organizational Commitment Scale (Mowday et al., 1979). Williams et al. (2016a) conducted a preliminary psychometric test of these scales in representing Fisher's model (*N* = 330) using exploratory factor analysis (EFA) and confirmatory factor analysis (CFA). Based on the results of the EFA and reliability analyses four items were removed from the battery, resulting in a final measure of 22 items, which were used here (α_t1_ = 0.95, α_t2_ = 0.86, α_t3_ = 0.91).

### Procedure

Participants completed a 3-day training course, which comprised ~6 h training contact time. Training materials were delivered via a mix of lecture and small group (2–4 people) activities, within groups of ~14–16 individuals. Two trainers facilitated each group, both of whom had received specialist training from the program authors to deliver the training materials. The program was based on materials developed by staff at the University of Pennsylvania's Positive Psychology Center and draws from the relevant theories and research underpinning the four elements of PsyCap. Examples from the training include: (1) participants are taught how to dispute negative thinking patterns with more optimistic perspectives, to foster optimism and hope; (2) participants learn about the ABC Model of cognitive behavioral therapy (Ellis, 1957) and how to identify deeply held beliefs that may be driving unhelpful thought patterns and behaviors to build resilience; and (3) at the end of each topic, participants identify how they could use the skill or knowledge taught in their personal and professional lives to build efficacy.

Participants were invited to complete a questionnaire at three time points: prior to day 1 of the training intervention (time 1), at the end of the final day of the intervention (time 2), and 8 weeks after the intervention ended (time 3). Completion of the survey was voluntary, which was outlined in a plain language statement provided to participants at time 1 and reinforced in the verbal preamble given by the researcher prior to the first survey. Before completing the survey at time 1, participants created a unique user name, which they used to complete the surveys again at times 2 and 3. This ensured anonymity whilst enabling the marrying of results across the three measurement occasions. The University of Melbourne's ethics review board approved all procedures.

The survey was available in hard copy at times 1 and 2. The responses were subsequently inputted manually by the researcher. At time 3 the survey was available online via the independent survey hosting website Survey Monkey (www.surveymonkey.com.au) and a link to the survey was sent to the participants via the research site's email system. The survey was available for a period of 10 days. Regular reminders were made throughout that time via email and verbal announcements at daily staff briefings to encourage participation.

### Analysis

Descriptive statistics and correlations were used to examine associations among explicit PsyCap, explicit OV, and work happiness across the three time points (hypotheses H1_a_ and H1_b_). This was then repeated for implicit PsyCap, implicit OV, and work happiness (H2_a_ and H2_b_).

Hypothesis 3 predicted that work happiness at time 3 (after the IRM process has time to work) would be highest when positive attitudes (PsyCap) are supported by perception of virtuousness in the organization culture (OV). Beginning with the explicit measures, participants who increased from time 2 to time 3 on both PsyCap and OV were coded as 1, and participants who decreased/stayed the same on one or both variables were coded as 0. This was repeated with the implicit measures. The mean work happiness levels at time 3 for the two groups were then compared using standardized *t*-tests.

Finally, based on the IRM, hypothesis 4 predicted that explicit and implicit attitudes of PsyCap and OV would follow the same pattern over time. Following the principles outlined by Barnes et al. (2012), a trend analysis was conducted for participants' levels of explicit and implicit PsyCap and OV. Specifically, for the four variables (explicit and implicit PsyCap and explicit and implicit OV), time 2 values were regressed on time 1 values, and time 3 values were regressed on time 2, using standard linear regression. Significantly positive beta weights indicate an increase from one time point to the next, and were coded as +1. Significantly negative beta weights indicate a decrease and were coded as −1. Non-significant changes were coded as 0. Then, a variable was created to indicate whether the pattern was the same for explicit and implicit variables (coded 1), or different (coded 0). For instance, if a participant increased from time 1 to time 2 and decreased from time 2 to time 3 on both the implicit and explicit PsyCap measures, they were coded as 1 for the final variable. A participant who increased then decreased on explicit PsyCap but increased and increased on implicit PsyCap was coded as 0. This coding was repeated for OV. An independent samples chi square tested whether there were a greater number of participants with the same pattern vs. those with a different pattern.

## Results

### Associations among psychological capital (PsyCap), organizational virtuousness (OV), and work happiness

Table 1 shows the means, standard deviations, and correlations amongst explicit PsyCap, implicit PsyCap, explicit OV, implicit OV, and work happiness, within and across the three measurement occasions. Supporting H1_a_, explicit PsyCap and OV were strongly positively correlated with one another, both cross-sectionally and over time, such that individuals with greater PsyCap perceived greater OV in the organization, and vice versa. Partially supporting H1_b_, explicit OV was strongly and significantly correlated with work happiness cross-sectionally and over time. Explicit PsyCap was significantly correlated with work happiness cross-sectionally, but was not significantly prospectively related to work happiness. H2_a_ was partially supported; correlations between implicit PsyCap and implicit OV were significant and positively associated within time but not prospectively. For example, correlation between time 2 implicit PsyCap and implicit OV was *r* = 0.91, whereas the correlation between time 2 implicit PsyCap and time 3 implicit OV was *r* = 0.13. In partial support of H2_b_, the associations between implicit PsyCap, implicit OV and work happiness within time were significantly positive at time 1 and time 3, but were not significant at time 2. Across time, implicit PsyCap, implicit OV, and work happiness were decreasingly correlated with one another.

**Table 1:** **Means, standard deviations and correlations for explicit and implicit PsyCap, explicit and implicit organizational virtuousness (OV), and work happiness within and across three measurement time-points**.

	**Variable**	***N***	***M***	***SD***	**1**	**2**	**3**	**4**	**5**	**6**	**7**	**8**	**9**	**10**	**11**	**12**	**13**	**14**	**15**
1	Explicit PsyCap Time 1	51	5.09	0.35	1.00													
2	Implicit PsyCap Time 1	52	1.55	0.93	0.11	1.00												
3	Explicit OV Time 1	50	5.38	0.54	0.36^**^	0.20	1.00												
4	Implicit OV Time 1	52	1.57	1.00	0.08	0.83^**^	0.19	1.00											
5	Work Happiness Time1	51	1.45	0.19	0.36^**^	0.11	0.56^**^	0.10	1.00										
6	Explicit PsyCap Time 2	51	5.24	0.39	0.72^**^	0.17	0.21	0.03	0.09	1.00									
7	Implicit PsyCap Time 2	52	2.07	0.83	0.25	0.01	0.02	0.18	−0.05	0.15	1.00								
8	Explicit OV Time 2	48	5.59	0.42	0.35^*^	0.21	0.61^**^	0.15	0.37^*^	0.44^**^	0.14	1.00							
9	Implicit OV Time 2	52	2.07	0.87	0.20	0.05	0.07	0.22	0.02	0.12	0.92^**^	0.17	1.00						
10	Work Happiness Time2	50	1.47	0.18	0.32^*^	0.11	0.44^**^	0.05	0.80^**^	0.26	−.05	0.54^**^	0.04	1.00					
11	Explicit PsyCap Time 3	38	5.07	0.40	0.36^*^	0.26	0.34^*^	0.01	0.33^*^	0.47^**^	−0.10	0.43^*^	−0.06	0.41^*^	1.00				
12	Implicit PsyCap Time 3	38	2.02	0.71	0.17	0.27	0.32	0.11	0.22	0.15	−0.11	0.23	−0.11	0.19	0.52^**^	1.00			
13	Explicit OV Time 3	38	5.26	0.47	0.14	0.20	0.50^**^	0.03	0.359^*^	0.11	0.07	0.42^*^	0.17	0.41^*^	0.63^**^	0.45^**^	1.00		
14	Implicit OV Time 3	38	1.98	0.75	0.25	0.21	0.42^**^	0.11	0.25	0.13	0.12	0.38^*^	0.13	0.27	0.36^*^	0.77^**^	0.63^**^	1.00	
15	Work Happiness Time3	38	1.53	0.13	0.05	0.03	0.17	−0.09	0.52^**^	0.15	0.05	0.44^**^	0.11	0.63^**^	0.46^**^	0.33^*^	0.50^**^	0.47^**^	1.00

*PsyCap, psychological capital; OV, organizational virtuousness. Work happiness is the average standardized scores on job satisfaction, engagement, and commitment, with a constant added to eliminate negative values*.

* *p < 0.05*,

** *p < 0.01*.

### The impact of positive PsyCap and OV attitudes on work happiness

Hypothesis 3 predicted that work happiness would be higher when positive PsyCap attitudes are supported by a positive evaluation of the organization environment (OV) than when they are not; this was partially supported. For the explicit measures, four participants (11%) increased in PsyCap and OV from time 2 to time 3, and 33 participants (89%) decreased in one or both variables. For the implicit measures, 13 participants (34%) increased in both implicit PsyCap and implicit OV from time 2 to time 3 and 25 (66%) decreased or stayed the same in one or both measures. The increase group had higher levels of work happiness than the decrease group for both the explicit measures (increase: *M* = 0.96, *SD* = 0.01; decrease: *M* = 0.93, *SD* = 0.07) and for the implicit measures (increase: *M* = 0.95, *SD* = 0.05; decrease: *M* = 0.93, *SD* = 0.08), although the difference was not statistically significant [explicit: *t*_(35)_ = 0.79, *p* = 0.08; implicit: *t*_(36)_ = 0.97, *p* = 0.16].

### Patterns of change in implicit and explicit attitudes

Finally, Hypothesis 4 predicted that implicit and explicit evaluations would have similar patterns of change over time. Table 2 shows the results of regressing each variable on the prior time point. On average, there were significant increases across the two time points in both explicit PsyCap and explicit OV, with no significant change in the implicit measures. Although the χ^2^ was significant [PsyCap: $\chi_{\left(1\right)}^{2}$ = 5.45, *p* = 0.02; or OV: $\chi_{\left(1\right)}^{2}$ = 6.81, *p* = 0.009], an examination of the pattern of results showed an opposite pattern, with a greater number of individuals having different patterns of change than the same. Only 18 participants had the same patterns of change for implicit and explicit PsyCap, and only 17 participants had the same patterns of change for implicit and explicit OV. Thus, hypothesis 4 was not supported.

**Table 2:** **Number of participants who increased/decreased in implicit and explicit PsyCap and OV from Time 1 to Time 2 and Time 2 to Time 3**.

**Time 1—Time 2**						**Time 2—Time 3**				
	***N***	**incr**	**decr**	**β**	***SE***	***N***	**incr**	**decr**	**β**	***SE***
**PsyCap**										
Explicit	49	37	12	0.77^**^	0.11^**^	37	13	24	0.44^**^	0.14^**^
Implicit	51	35	16	−0.06	0.13	38	19	19	−0.08	0.12
**OV**										
Explicit	46	38	8	0.47^**^	0.09^**^	34	10	24	0.54^*^	0.20^*^
Implicit	51	37	14	0.08	0.12	38	16	22	0.12	0.13

*PsyCap, psychological capital; OV, organizational virtue, incr., number whose score increased across the time period; decr., number whose score decreased across the time period, SE, standard error*.

* *p < 0.05*,

** *p < 0.01*.

In summary, the results provide some preliminary support for hypotheses 1, 2, and 3. Hypotheses 1 and 2 explored the associations between implicit and explicit attitudes of PsyCap and OV, and work happiness. Overall, there were significant relationships between the variables within time, but less so over time. Hypothesis 3 examined whether work happiness was higher when PsyCap attitudes are supported by a positive view of the organization culture (OV). The results followed the expected pattern but were not statistically significant. Finally, hypothesis 4 tested the proposition made in the IRM that implicit and explicit attitudes would have similar patterns of change over time. This was not supported.

## Discussion

This study examined the Iterative-Reprocessing Model (IRM) of evaluation as a process underlying the associations between positive employee attitudes (PsyCap), perception of positive organization culture (OV), and work happiness. Notably, employee PsyCap, OV, and work happiness were associated through implicit *and* explicit evaluation processes. This supports the call for examination of non-conscious processes such as implicit attitudes in organizational research (Latham et al., 2010). Results also suggest that the IRM is a promising model for future researchers seeking to understand associations amongst inside factors and outside factors that influence employee work happiness. Moreover, they provide additional support that both inside-out and outside-in factors influence work happiness, that increased PsyCap enables employees to evaluate virtues in their work environment more easily (Williams et al., 2015) and for the dual intervention approach proposed by the IO-OI model (Williams et al., 2016b). Practically, this research highlights the value of fostering positive employee attitudes *at* work as well as positive attitudes toward work to foster work happiness and emphasizes the need for outcomes from positive employee development programs to be supported and reinforced by organizational culture.

### Inside and outside predictors of work happiness

The IO-OI model suggests that factors both inside and outside employees impact their well-being. In the current study, inside and outside factors were operationalized as PsyCap and OV, respectively. Within and across time, PsyCap and OV were positively associated with one another. As expected, OV correlated with work happiness cross-sectionally and over time. However, PsyCap was only correlated with work happiness at time 3.

The timing of the intervention on the work calendar may provide insight into the lack of association between work happiness and PsyCap until the final assessment. Williams et al. (2015) propose that PsyCap attitudes enable and support employee work happiness as it provides them with the personal resources to perform well in their work role. However, the training intervention was held at the end of an extended holiday period, such that prior to time 1, much of the current sample had been away from their work role for ~10 weeks. As such, the sample did not have an opportunity to apply positive PsyCap attitudes in the workplace until after the intervention finished. By time 3, the sample had returned to work for 8 weeks and, therefore, they had the opportunity to access positive PsyCap attitudes *at work*. By the end of this 8 week period, a positive significant association was found between PsyCap and work happiness. This suggests that training may only be effective if employees have the opportunity to put the training into practice in their work role. It might also suggest that there is a time-lag process for people to take what they learn in training and put it into practice at work. Future studies should consider the timing of interventions and the time-lag effect of transfer of training as a potential confound.

### The iterative reprocessing of attitudes and positive attitude development

The Iterative-Reprocessing Model (IRM) suggests that implicit evaluation processes influence and are influenced by explicit reflective processes in an ongoing evaluative cycle (Cunningham and Zelazo, 2007; Van Bavel et al., 2012). The model provides an organizing framework for leaders to understand how to develop positive attitudes in their employees as automated (implicit) and reflective (explicit) attitudes collaboratively work together (Van Bavel et al., 2012). While the current findings support the value of including implicit and explicit attitudes in employee wellbeing research, there was not support for parallel processes occurring at implicit and explicit levels. While the model suggests that the two levels would co-occur, more participants had different patterns than the same.

There are several possible explanations for this pattern of results. It may indeed be that the model mis-specifies the patterning of explicit and implicit attitudes, or that the processes operate independently yet in parallel (Fazio and Olson, 2003; Gawronski and Strack, 2004). Alternatively, the timing of measures used in the study may have been insufficient for capturing the dual process. Cunningham and Zelazo (2007) propose that the iterative cycles of evaluation take place in milliseconds, whereas the current study considered much longer time scales (3 days and 8 weeks). Further consideration of the time course of the IRM is needed.

The IRM further suggests that attitude development and change is influenced by a pre-appraisal process, contextual information, and motivational factors (Van Bavel et al., 2012). Based on this, it was expected that when increases in PsyCap are supported and reinforced by positive context and motivational cues in the organizational environment (OV), higher levels of employee happiness at work ensue. Although the pattern of results followed this expectation, associations were not significant. The small sample size limits the power to find such a pattern. Thus, while the hypothesis proposed is not supported, the pattern of results suggests that further research in this area is worthwhile.

### Implicit and explicit attitudes

A particular strength of the study is that it included both explicit and implicit measures, responding to the call for examination of non-conscious processes such as implicit attitudes in organizational research (Latham et al., 2010) and specifically in relation to organization culture (Uhlmann et al., 2012).

Across time, patterns of association amongst implicit PsyCap, implicit OV, and work happiness matched the explicit ones, in that variables closer in time were more strongly correlated, and correlation strength declining over time. McClelland et al. (1995) proposed that implicit processes need several experiences to produce an aggregate knowledge base; as such they are slower to change and are more stable. The weakening association between implicit attitudes over time in the current study challenges this, and provides support for Van Bavel et al.'s (2012) proposition that implicit attitudes are as open to change as explicit ones. It also suggests that implicit measures access different aspects of employee attitudes to explicit ones.

Many phenomena of interest to researchers operate outside of an employee's conscious awareness and control and various studies have illustrated the limited accuracy of conscious reflection through the difficulty people have identifying influencing factors in their attitudes and decisions (e.g., Nisbett and Wilson, 1977; Wilson, 2002). There are a number of reasons for the need to understand and measure non-conscious processes such as implicit attitudes. First, the knowledge-based high-cognitive load work in organizations likely means that that implicit processing is extensive in organizational life (Johnson and Steinman, 2009) and as such is worthy of study. Second, a range of important organizational outcomes such as leadership performance, creativity and employee health outcomes have been associated with implicit psychological constructs (Schultheiss, 2008).

Third, as results of the current study and other research shows, there is evidence that explicit and implicit attitudes can explain unique variance in outcomes of interest and are not necessarily correlated (McClelland et al., 1989; Bing et al., 2007). This suggests that there may be an additive influence of implicit attitudes (and other implicit constructs) on outcomes of interest. Fourth, implicit attitudes are processed in the automatic somatic and affective systems of the brain (Lieberman, 2007) and so are less susceptible to problems with self-report measures such as socially desirable responding (Roberts et al., 2006) and can change more quickly. As such, it may be that the implicit measures provide a timelier reflection of attitude change. Further, the projective nature of many implicit measures may access changes in self-concept more quickly and be less dependent on changing behavior, such that implicit measures may be more effective in capturing the immediate impact of interventions. This provides a strong rationale for future organizational research to include both explicit and implicit measures.

### Study limitations and strengths

The results of this study need to be considered within a number of limitations. First, the outcome variable, work happiness, was based on Fisher's (2010) model, but there is not a single validated measure to date that measures work happiness as a construct comprising engagement, commitment, and satisfaction. The current study used a refined measure from previous research (Williams et al., 2016a), which combined three psychometrically validated measures for the three inter-related domains proposed by the model. Further testing of Fisher's model and a psychometrically strong measure of the model is needed.

Second, self-report measures were used, such that associations might reflect common source bias rather than true effects. The current study added two implicit measures, but incorporating other approaches and objective measures (e.g., customer experience surveys and levels of employee absenteeism), will be useful. Third, the implicit OV measure was developed specifically for this study. The measure mirrored the I-PCQ (Harms and Luthans, 2012), and initial psychometrics of the measure were adequate, but a full test of the measure was beyond the scope of this study. Further, development of a validated implicit OV measure is needed. Fourth, the sample size was small, which limited the analyses possible. Finally, the study was conducted with employees who work in a school, which may not be generalizable to workers from other sectors.

Despite these limitations, the study makes several valid contributions. By investigating the attitudinal processes underlying the interrelationships between PsyCap, perception of organizational virtues, and work happiness with a sample of school employees, the current study integrates aspects of the fields of attitudes, positive organizational scholarship, positive organizational behavior, and positive education. It is one of the first studies to apply the theory of the IRM to explain associations amongst PsyCap, OV, and work happiness, to test the IRM theory in a field-based setting and, to examine how PsyCap, OV, and work happiness interrelate through changes in explicit and implicit attitudes over time. The intervention-based design of the study provides valuable understanding of the patterns of the associations between PsyCap, OV, and work happiness when PsyCap is intentionally targeted for development and it provides support for the dual-approach to developing work happiness proposed by the Inside-out Outside-in model. Further, it responds calls from the field including the need to explore the influence of implicit attitudes in the workplace (Uhlmann et al., 2012) and the need to understand more about the underlying mechanisms of positive psychology interventions (Lyubomirsky and Layous, 2013).

## Conclusion

There is increasing evidence that employee happiness at work influences positive outcomes for both employees and organizations. As such, wellbeing has become an important area of focus in positive organizational research. This study highlights the merit in understanding non-conscious processes such as implicit attitudes in wellbeing research, and their value to positive psychology interventions measurement programs. Further, the understanding of attitudinal processes underlying changes in work happiness that comes from the study provides an important foundation for effectively building and supporting employee wellbeing. It is hoped that attitudes receive considerably more attention in workplace wellbeing research in the future.

## Ethics statement

This study was approved by the Melbourne Graduate School of Education Ethics Committee. Participants were informed about the research project in an email from the CEO of the research site. This was supported by an email from the primary researcher inviting them to participate in the research with a Plain Language Statement as an attachment. Participants were then asked to complete a Consent Form at the first survey time-point.

## Author contributions

PW, MK, and LW: made substantial contributions to the conception or design of the work or the acquisition, analysis, or interpretation of data for the work; were involved with drafting the work or revising it critically for important intellectual content; have given final approval of the version to be published; agree to be accountable for all aspects of the work in ensuring that questions related to the accuracy or integrity of any part of the work are appropriately investigated and resolved.

### Conflict of interest statement

PW was employed at the research site at the time of the study. The other authors declare that the research was conducted in the absence of any commercial or financial relationships that could be construed as a potential conflict of interest.
