# Supplementary material for: The Role and Reprocessing of Attitudes in Fostering Employee Work Happiness: An Intervention Study
Source: Front Psychol. 2017 Jan 19;8:28. doi: 10.3389/fpsyg.2017.00028 (PMC5243841; doi:10.3389/fpsyg.2017.00028)
Supplement: Supplementary file 1 [file DataSheet1.DOCX]

**Supplementary Information**

**Development and initial psychometric information for the implicit organizational virtues measure**

While there is an existing measure of implicit PsyCap (I-PCQ) attitudes, there is not an existing measure of implicit attitudes of organizational virtue (OV). We sought advice from Peter Harms (co-author of the I-PCQ) to construct an implicit OV (i-OV) measure. The resulting measure uses the stories and framework of the I-PCQ, with participants rating each story for the five organizational virtues rather than the four PsyCap factors.

The I-PCQ (Luthans & Harms, 2012) uses a semi-projective technique with written prompts followed by short questions. Respondents are presented with three situational prompts and asked to imagine stories relating to the prompt about a fictional character (not themselves). They are then asked to respond to construct-targeted questions about the stories they generated. The three prompts are based in an organizational context with one presenting a positive experience (“Someone has a new job”), one a negative experience (“Someone makes a mistake at work”), and the third an ambiguous experience (“Someone talks to their supervisor”).

For each of the stories, participants indicated on a 7-point Likert scale how much each OV factor represents the character (-3 = the opposite is very true of this character, 0 = irrelevant thought/feeling for this character, and +3 = very true of this character).

For each of the stories, the five factors of OV were assessed by two items, which aligned with two of the three explicit items. Table A1 indicates the implicit OV items used for each prompt.

Each time point thus had 6 items assessing each of the five domains. As indicated in Table A2, reliability for each domain was adequate. The resulting 10 items across the three prompts were averaged together to represent an individual’s implicit OV (30 items, α_t1_ = .96, α_t2_ = .98, α_t3_ = .97).

**Table A1**

*Items from the Organizational Virtuousness Scale (Cameron, Bright, & Caza, 2004), with the implicit version used for the i-OV.*

| **OV element** | **Explicit OV items** | **Implicit OV items** |
| --- | --- | --- |
| **Optimism** | A sense of profound purpose is associated with what we do here | A sense of profound purpose is associated with what we do here |
|  | In this organisation we are dedicated to doing good in addition to doing well | -- |
|  | We are optimistic that we will succeed, even when faced with major challenges | We are optimistic that we will succeed, even when faced with major challenges |
| **Trust** | Employees trust one another in this organisation | *--* |
|  | People are treated with courtesy, consideration, and respect in this organisation | People are treated with courtesy, consideration, and respect in this organisation |
|  | People trust the leadership of this organisation | People trust the leadership of this organisation |
| **Compassion** | Acts of compassion are common here | Acts of compassion are common here |
|  | This organisation is characterised by many acts of caring and concern for other people | This organisation is characterised by many acts of caring and concern for other people |
|  | Many stories of compassion and concern circulate among organisation members | -- |
| **Integrity** | Honesty and trustworthiness are hallmarks of this organisation | Honesty and trustworthiness are hallmarks of this organisation |
|  | This organisation demonstrates the highest levels of integrity | This organisation demonstrates the highest levels of integrity |
|  | This organisation would be described as virtuous and honourable | *--* |
| **Forgiveness** | We try to learn from our mistakes here, consequently missteps are quickly forgiven | *--* |
|  | This is a forgiving compassionate organisation in which to work | This is a forgiving compassionate organisation in which to work |
|  | We have very high standards of performance, yet we forgive mistakes when they are acknowledged and corrected | We forgive mistakes when they are acknowledged and corrected |

**Table A2**

*Reliability (Cronbach’s a) for the five OV factors, for each time point.*

|  | **Time 1**  *(n = 55)* | **Time 2**  *(n = 55)* | **Time 3**  *(n = 43)* |
| --- | --- | --- | --- |
| **Optimism** | 0.83 | 0.91 | 0.9 |
| **Trust** | 0.83 | 0.9 | 0.88 |
| **Compassion** | 0.85 | 0.91 | 0.87 |
| **Integrity** | 0.87 | 0.9 | 0.91 |
| **Forgiveness** | 0.81 | 0.92 | 0.87 |
